# An Inducible CYP19A1 Excision Model for Sexual Differentiation in Chicken (*Gallus gallus*) via the CRISPR/Cas9 System

**DOI:** 10.3390/vetsci12040296

**Published:** 2025-03-22

**Authors:** Guangzheng Liu, Yixiu Peng, Zeyu Li, Xilin Zhu, Hongyan Sun, Guohong Chen, Qisheng Zuo, Yingjie Niu, Jiuzhou Song, Wei Han, Wanhong Wei, Bichun Li, Kai Jin

**Affiliations:** 1 Joint International Research Laboratory of Agriculture and Agri-Product Safety, Ministry of Education of China, Yangzhou University, Yangzhou 225009, China; dx120230169@stu.yzu.edu.cn (G.L.); pyx3450@163.com (Y.P.); mx120230901@stu.yzu.edu.cn (Z.L.); mz120231580@stu.yzu.edu.cn (X.Z.); hongyans2392@163.com (H.S.); ghchen2019@yzu.edu.cn (G.C.); 006664@yzu.edu.cn (Q.Z.); niuyj@yzu.edu.cn (Y.N.); 2 Key Laboratory of Animal Breeding Reproduction and Molecular Design for Jiangsu Province, College of Animal Science and Technology, Yangzhou University, Yangzhou 225009, China; 3 Institutes of Agricultural Science and Technology Development, Yangzhou University, Yangzhou 225009, China; 4 Department of Animal & Avian Sciences, University of Maryland, College Park, MD 20742, USA; songj88@umd.edu; 5 Jiangsu Institute of Poultry Sciences/Poultry Institute, Chinese Academy of Agricultural Sciences, Yangzhou 225003, China; hanwei830@163.com; 6 College of Bioscience and Biotechnology, Yangzhou University, Yangzhou 225009, China; whwei@yzu.edu.cn; 7 College of Biotechnology, Jiangsu University of Science and Technology, Zhenjiang 212100, China

**Keywords:** chickens, gene knockout, gender control, CRISPR/Cas9, Tet-on

## Abstract

Aromatase, a crucial enzyme for estrogen synthesis, plays a vital role in gender determination and differentiation. This study aimed to establish an inducible knockout model of the chicken *CYP19A1* gene, which encodes aromatase, to support gender control in chickens. We selected the most efficient sgRNA target site and constructed an inducible knockout model based on the Tet-on system. The knockout efficiency reached 80% with 20 μg/mL DOX induction in vitro. The encapsulation of the plasmid with PEI and injection into eggs achieved a knockout efficiency of 45% in ovo. qRT-PCR analysis revealed a significant downregulation of female-related genes (*CYP19A1*, *FOXL2*, *ESR1*) and upregulation of male-related genes (*DMRT1*, *SOX9*, *AMH*) in female chicken embryos after induction. Western blotting showed decreased protein expression of CYP19A1 and FOXL2, and increased SOX9 expression in female embryos post-DOX induction. Elisa detection further confirmed lower estradiol levels in the gonads of induced female embryos compared to normal and non-induced females. These findings demonstrate the successful establishment of an inducible knockout system for the *CYP19A1* gene in chickens, providing theoretical and technical support for the creation of new breeding materials for gender control.

## Simple Summary

In this study, we created a special method to control the sex of chickens by targeting a vital enzyme called aromatase, which is important for making estrogen. We built a model where the aromatase gene in chickens could be turned off by using a dox-inducible system. We demonstrated that when this system is used, the female-related genes were reduced and the male-related genes were increased in female chicken embryos. These results show that the stargates could change the sexual characteristics of chicken embryo gonads, providing a protentional to build breeding materials for sexual control in chickens.

## 1. Introduction

The economic significance of gender determination in chickens is particularly evident in the layer industry, where only females are economically viable. This results in the culling of male chicks at birth, which has significant waste implications and raises ethical concerns [1,2]. Statistical data indicate that the annual economic loss resulting from the culling of male chicks may exceed 7 billion dollars [3]. Current methods for sex determination in poultry production, such as vent sexing, feather color and feather growth rate, laser detection of egg contents, and DNA sequence differences, require substantial manpower, resources, and financial investment, making them unsuitable for modern farming requirements [4,5,6,7,8]. Therefore, there is an urgent need for effective methods of sex determination and control to reduce production costs and ensure the welfare of poultry.

Cytochrome P450 family 19 subfamily A member 1 (*CYP19A1*) is a crucial enzyme in estrogen synthesis (17β-estradiol, E2) in most vertebrates, and estrogen regulates gender differentiation in amphibians, fish, reptiles, birds, and mammals [9,10,11,12]. Studies have shown that the development of undifferentiated gonads in birds into testes or ovaries depends on the activity of aromatase [13]. Inhibition of aromatase activity by inhibitors suppresses estrogen synthesis, preventing ovarian formation in chicken embryos and promoting gonadal development towards testes, with the degree of sex reversal depending on the intensity of inhibitor action [14]. The overexpression of *CYP19A1* in male chicken embryos results in enlarged left gonads with ovarian structural development, and key genes involved in male gonadal development are inhibited [15]. Therefore, the *CYP19A1* gene is crucial in avian embryonic development for gender differentiation, and its genetic modification provides new material for chicken gender control.

Meanwhile, the development of CRISPR/Cas9 technology offers an effective method for gene editing [16]. Yosef et al. utilized CRISPR/Cas9 technology to introduce three knockout targets regulating early development of the Y chromosome of male mice, resulting in blocked development of male embryos and exclusive production of female offspring when crossed with females carrying Cas9 on autosomes [17]. Stephanie et al. also utilized CRISPR/Cas9 technology to establish a transgenic line expressing Cas9 on the Y chromosome in fruit flies, which can be used to control pests in XY sex systems [18]. Further research has shown that the Tet-on inducible transgenic expression system can be used to regulate the expression of Cas9 or sgRNA to produce inducible CRISPR/Cas9 systems [19,20]. Lu et al. utilized a doxycycline-inducible Tet-on system to regulate Cas9 expression and simultaneously target two oncogenic lncRNAs to inhibit the spread of bladder cancer cells [21]. Meiying et al. combined CRISPR/Cas9 and Tet-on systems to construct an inducible TLE6 protein knockout (KO) mouse spermatogonial cell line to investigate the effect of Tle6 on spermatogonial cell proliferation [22]. Thus, CRISPR/Cas9 and Tet-on systems are available to directed control in the cellular and organismal levels.

Previous studies have successfully combined the Tet-on inducible system with in ovo electroporation, enabling spatiotemporal control of specific gene expression in chicken embryos [23]. However, methods for sex control in chickens remain under exploration, while gene editing technologies have demonstrated potential in this area. Therefore, the objective of this study is to establish an inducible knockout model utilizing the Tet-on system, targeting the chicken *CYP19A1* gene, which encodes aromatase, to achieve precise sex control in chickens. Thus, this study successfully developed an inducible CRISPR/Cas9-mediated knockout system targeting the *CYP19A1* gene in chickens by integrating the CRISPR/Cas9 system with Tet-on technology. This achievement lays a crucial foundation for the subsequent insertion of sex chromosomes, facilitating the construction of an artificially controllable model for chicken sex regulation.

## 2. Materials and Methods

### 2.1. Ethics Statement

Fresh, fertilized, and unhatched eggs of Rugao yellow chickens were obtained from the farm of the Poultry Research Institute (Chinese Academy of Agricultural Science, Yangzhou, China). All procedures involving the care and use of animals conformed to the U.S. National Institute of Health guidelines (NIH Pub. No. 85-23, revised 1996) and were approved by the Laboratory Animal Management and Experimental Animal Ethics Committee of Yangzhou University, China (202203074).

### 2.2. Construction of CYP19A1 Knockout Vector

Three sgRNA target sites, sgRNA1, sgRNA2, and sgRNA3, were designed based on the coding sequence (CDS) region of *CYP19A1* in the NCBI database. The target sequences can be found in Table 1. Oligo primers of *CYP19A1*-1, *CYP19A1*-2, and *CYP19A1*-3 were synthesized according to the target sequences and annealed to form double-stranded DNA, which was then ligated into the vector VK001-08. Following the instructions of the TreliefTM 5α Chemically Competent Cell Kit (Tsingke, Beijing, China), the vector and competent cells were incubated on ice for 5 min, heat-shocked at 42 °C for 45 s, chilled on ice for 2 min, plated on solid culture medium containing ampicillin, and incubated at 37 °C for 16 h. Single colonies were picked for further analysis. Plasmid extraction was performed using the EndoFree Maxi Plasmid Kit (Tiangen, Beijing, China) according to the manufacturer’s instructions, and the primer sequences are listed in Table 2.

### 2.3. Construction of the TRE-EGFP Vector

The primer of EGFP-1 was designed based on the sequence of the EGFP element in pEGFP-N1, incorporating EcoRI and BamHI restriction sites. Using the PrimeSTAR Max DNA Polymerase Kit (TaKaRa, Dalian, China), the EGFP element containing the restriction sites was amplified from the pEGFP-N1 plasmid template. The amplified product was digested with EcoRI and BamHI restriction enzymes (NEB, Ipswich, MA, USA) according to the manufacturer’s instructions, followed by agarose gel electrophoresis on a 1% gel. The desired band was excised and purified using the Universal DNA Purification Kit (Tiangen, Beijing, China). The digested amplicon and TRE-tight vector were ligated using the T4 DNA Ligase Kit (NEB, Ipswich, MA, USA) at 16 °C for 16 h. The ligation product was transformed into competent cells following the instructions of the TreliefTM 5α Chemically Competent Cell Kit (Tsingke, Beijing, China), and single colonies were picked for further analysis. The primer sequences are provided in Table 2.

### 2.4. Construction of TRE-Cas9-rtTA-Puro and sgRNA-sy004 Vectors

An inducible *CYP19A1* knockout system was constructed by constructing two separate vectors for Cas9 and sgRNA components. One vector contained an inducible Cas9 cassette, while the other contained a single sgRNA cassette. Both vectors were co-transfected, and induction with DOX (Solarbio, Beijing, China) facilitated the knockout of *CYP19A1*. The primer of Puro-1 was designed based on the sequence of the Puro element in vector VK001-08, incorporating EcoR and XbaI restriction sites. The Puro element containing the restriction sites was amplified by PCR using the VK001-08 plasmid as a template. Primers Puro+rtTA-1 and Cas9-1 were designed based on the sequences of the Puro-rtTA element in pTet-on-Advanced-Puro and the Cas9 element in VK001-08, respectively, introducing the KpnI, NotI, and PciI restriction sites. The Puro-rtTA element containing the restriction sites was amplified from the pTet-on-Advanced-Puro plasmid, while the Cas9 element containing the restriction sites was amplified from the VK001-08 plasmid. These elements were then assembled to construct the TRE-Cas9-rtTA-Puro vector. sgRNA2 was ligated into the sy004 vector to construct the sgRNA-004 vector, which is named *CYP19A1*-sgRNA. The primer sequences are provided in Table 2.

### 2.5. Cell Transfection

DF-1 and CEF cells were adjusted to a density of 1 × 105 cells/mL and cultured until reaching 80% confluency. According to the instructions of the Lipo8000™ Transfection Reagent Kit (Beyotime, Shanghai, China), 1 μg of plasmid and 1.5 μL of transfection reagent were mixed in 25 μL of Opti-MEM™ I Reduced Serum Medium (Thermo Fisher Scientific, Waltham, MA, USA). The mixture was evenly added to the cells, and fluorescence observation and sample collection were conducted 48 h post-transfection.

### 2.6. RNA Isolation and Reverse Transcription

A total of 1 × 106 cells were collected and lysed with 1 mL of Trizol reagent (Vazyme, Nanjing, China). After incubation at room temperature for 5 min, an equal volume of chloroform (Tiangen, Beijing, China) was added. The samples were vigorously shaken for 15 s, followed by a 3 min incubation at room temperature and centrifugation at 10,000 rpm for 15 min at 4 °C. Isopropanol was added to the aqueous phase, and the mixture was incubated for 10 min. After centrifugation at 10,000 rpm for 10 min at 4 °C, the supernatant was discarded. The RNA pellet was washed with 75% ethanol, followed by centrifugation at 10,000 rpm for 5 min at 4 °C, and the supernatant was discarded. The RNA concentration was determined using a NanoDrop 2000 spectrophotometer (Thermo Fisher Scientific, Waltham, MA, USA).

Reverse transcription of RNA was performed using the HiScript^®^ III RT SuperMix (+gDNA) Kit (Vazyme, Nanjing, China). The reaction mixture consisted of 1 μg of RNA, 4 μL of 4× gDNA Wiper Mix, and ddH_2_O, reaching a final volume of 16 μL. The mixture was incubated at 42 °C for 2 min, followed by the addition of 4 μL of 5× HiScript III qRT SuperMix. The synthesis of cDNA was performed using the following PCR program: 37 °C for 15 min, followed by 85 °C for 5 s.

### 2.7. qRT-PCR

According to the instructions of the ChamQ Universal SYBR qPCR Master Mix Kit (Vazyme, Nanjing, China), the reaction mixture comprised 10 μL of 2× ChamQ Universal SYBR qPCR Master Mix, 2 μL of cDNA, 0.4 μL of forward primer, 0.4 μL of reverse primer, and ddH_2_O, reaching a final volume of 20 μL. The qPCR program consisted of an initial denaturation at 95 °C for 30 s, followed by 40 cycles of denaturation at 95 °C for 10 s, annealing at 60 °C for 30 s, and extension at 72 °C for 15 s, with a final extension at 72 °C for 5 min. The genes detected included *AMH*, *SOX9*, *FOXL2*, *ESR1*, *CYP19A1*, *DMRT1*, *Cas9*, and *PURO*, with *β-ACTIN* used as the reference gene. The primer sequences for amplifying each gene are listed in Table 2.

### 2.8. Western Blot (Supplementary Materials)

Cells and tissues were lysed by adding RIPA buffer (Tiangen, Beijing, China) on ice for 20 min, and the protein concentration was measured. Twenty micrograms of protein samples were mixed with 6× SDS PAGE loading buffer (Beyotime, Shanghai, China) and denatured at 100 °C for 10 min. Electrophoresis was performed at 80 V for 10 min, followed by 120 V for 40–80 min, and PVDF membranes (Solarbio, Beijing, China) corresponding to the desired sizes were excised. The membranes were immersed in transfer buffer for 15 min and then transferred at 11 V for 23 min. The PVDF membranes were blocked in 5% skim milk (Wako, Shanghai, China) for 2 h and washed with TBST (Solarbio, Beijing, China). Primary antibodies (GFP, Poteintec, Wuhan, 1:200,000, 66002-1-Ig; FOXL2, Poteintec, Wuhan, 1:1000, 19672-1-AP; SOX9, Poteintech, Wuhan, 1:10,000, 67439-1-IG; CYP19A1, Bio-Rad, Bejing, 1:5000, MCA2077; Cas9, Abcam, Shanghai, 1:5000, ab189380) were incubated overnight at 4 °C. Secondary antibodies (Goat Anti-Mouse IgG, CWBIO, Taizhou, 1:2000, CW0102S; Goat Anti-Rabbit IgG, CWBIO, Taizhou, 1:2000, CW0103S) were applied for 2 h, followed by washing and visualization using the ECL method [24].

### 2.9. Luciferase-SSA Activity Assay

The sgRNA sites were inserted into the Luciferase region of the Luciferase-SSA vector upstream of the Luciferase terminator, constructing the SSA reporter vector. Cells were divided into the following two groups for transfection: one group received the reporter vector, knockout vector, and donor vector, while the other group received an empty vector. After 48 h, cells were collected, and the Luciferase expression activity was measured using a luminometer.

### 2.10. T7E1 Enzyme Digestion Assay

Genomic DNA was extracted from the transfected cells at a density of 1 × 106 cells/mL using the TIANamp Genomic DNA Kit (Tiangen, China). The extracted DNA served as a template for PCR amplification of the knockout fragment before and after approximately 500 bp, according to the instructions of the PrimeSTAR Max DNA Polymerase Kit (TaKaRa, Dalian, China). Primers *CYP19A1*sg1, *CYP19A1*sg2, and *CYP19A1*sg3 were designed for amplification, and their sequences are listed in Table 1. The PCR products were then subjected to T7 Endonuclease I digestion according to the instructions of the T7 Endonuclease I Kit (NEB, Ipswich, MA, USA). The reaction conditions included the initial denaturation at 95 °C for 10 min, followed by a touchdown PCR program, as follows: starting from 95 °C for 2 s and decreasing by 0.1 °C every 2 s until 75 °C, then decreasing by 0.1 °C every second until 16 °C, followed by incubation at 16 °C for 2 min, and, finally, digestion at 37 °C for 30 min. The digested products were analyzed by 1% agarose gel electrophoresis for observation and quantification of the results.

### 2.11. TA Cloning

Following the instructions of the 5 min TA/Blunt-Zero Cloning Kit (Vazyme, Nanjing, China), DNA fragments of approximately 500 bp before and after the knockout region were amplified by PCR and ligated into the T vector. The ligated products were then transformed into competent cells using the TreliefTM 5α Chemically Competent Cell Kit (Tsingke, Beijing, China). Fifteen individual colonies were picked from each LB agar plate and subjected to sequencing analysis.

### 2.12. PEI-Mediated Injection

The experiment was divided into two groups, designated as A and B. In Group A, 12 μg of plasmid per egg was mixed with 20 μg/μL DOX and PBS, reaching a final volume of 40 μL per egg. In Group B, 18 μg of PEI per egg was mixed with PBS, reaching a final volume of 60 μL per egg. The solution from Group A was added to Group B and mixed thoroughly by shaking. After incubating at 37 °C for 15 min, the mixture was injected into the blunt end of eggs in an egg incubator chamber. The eggs were then sealed with paraffin and placed in an incubator for hatching.

### 2.13. ELISA Detection

Gonads were collected from the female chicken embryos at the developmental stages of 4.5, 6.5, 8.5, 10.5, 12.5, 14.5, 16.5, and 18.5 days to detect the estradiol expression level. Tissue samples were homogenized in 200 μL PBS using a homogenizer to prepare cell suspensions. After preparing the standard solution, 100 μL of the standard solution, 10 μL of the sample, and 90 μL of biotin detection antibody were added to an enzyme-linked immunosorbent assay (ELISA) plate and incubated at 37 °C for 30 min. After washing the plate 5 times with washing solution and blotting dry on filter paper, 100 μL of the SABC (Streptavidin-Biotin Complex) compound was added and incubated at 37 °C for 20 min. Following the washing steps, 100 μL of the substrate working solution was added, and it was incubated at 37 °C in the dark for 15 min [25]. Subsequently, 50 μL of stop solution was added, and the absorbance was measured at 450 nm using a microplate reader (Tecan, Männedorf, Switzerland).

### 2.14. Statistical Analysis

All experiments were conducted more than three times, and representative images are shown in the figures. Statistical analysis was performed using the one-way analysis of variance (ANOVA) or Student’s *t*-test. Prior to analysis, all percentage data were subject to arcsine transformation, and data were presented as mean ± SEM. *p* < 0.05 was considered statistically significant. * *p* < 0.05; ** *p* < 0.01; *** *p* < 0.001; **** *p* < 0.0001.

## 3. Results

### 3.1. Establishment of CYP19A1 Knockout System In Vitro

**Figure 1:**
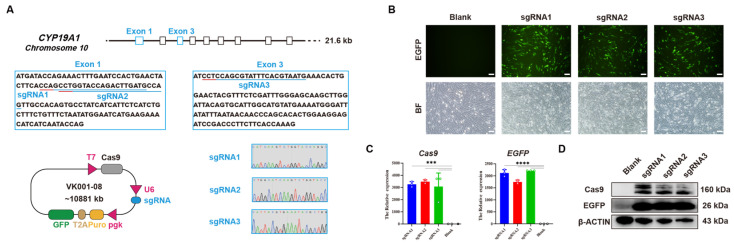
Establishment of CRISPR/Cas9-mediated *CYP19A1* knockout system in vitro. (**A**) Schematic diagram of the *CYP19A1* knockout target site and schematic diagram of the knockout vector and sequencing peaks. Blue underline: Targeted sequence sites for experimental design. Red underline: Protospacer Adjacent Motif (PAM). (**B**) Fluorescence image of cells transfected with *CYP19A1* knockout vector. (**C**) Quantitative expression of *Cas9*, *EGFP* in *CYP19A1* knockout vector. (**D**) *CYP19A1* knockout vector protein expression of Cas9, EGFP. (*** *p* < 0.001; **** *p* < 0.0001).

The target sites sgRNA1 and sgRNA2 were designed on the first exon, and target site sgRNA3 was designed on the third exon of the chicken *CYP19A1* gene (Gene ID: 414854) via bioinformatics tools available on the NCBI database (Figure 1A). Oligos synthesized by the company were annealed to form double strands and ligated into the vector VK001-08. Sequencing alignment results confirmed the successful construction of vectors *CYP19A1*-sgRNA1, *CYP19A1*-sgRNA2, and *CYP19A1*-sgRNA3 (Figure 1A). DF-1 cells were transfected with the constructed *CYP19A1* knockout vectors, and after 48 h of transfection, fluorescence expression was observed (Figure 1B). qRT-PCR analysis of Cas9 and EGFP expression revealed a significant increase in Cas9 and EGFP expression levels in cells transfected with the *CYP19A1*-sgRNA1, *CYP19A1*-sgRNA2, and *CYP19A1*-sgRNA3 knockout vectors compared to the control group. Western blotting (WB) results also showed the expression of Cas9 and EGFP proteins in cells transfected with *CYP19A1*-sgRNA1, *CYP19A1*-sgRNA2, and *CYP19A1*-sgRNA3, while no expression was observed in the blank control (Figure 1D). These results suggested that we successfully constructed the *CYP19A1* knockout vectors and they can normally express in cells.

### 3.2. Identification of CYP19A1 Knockout System

**Figure 2:**
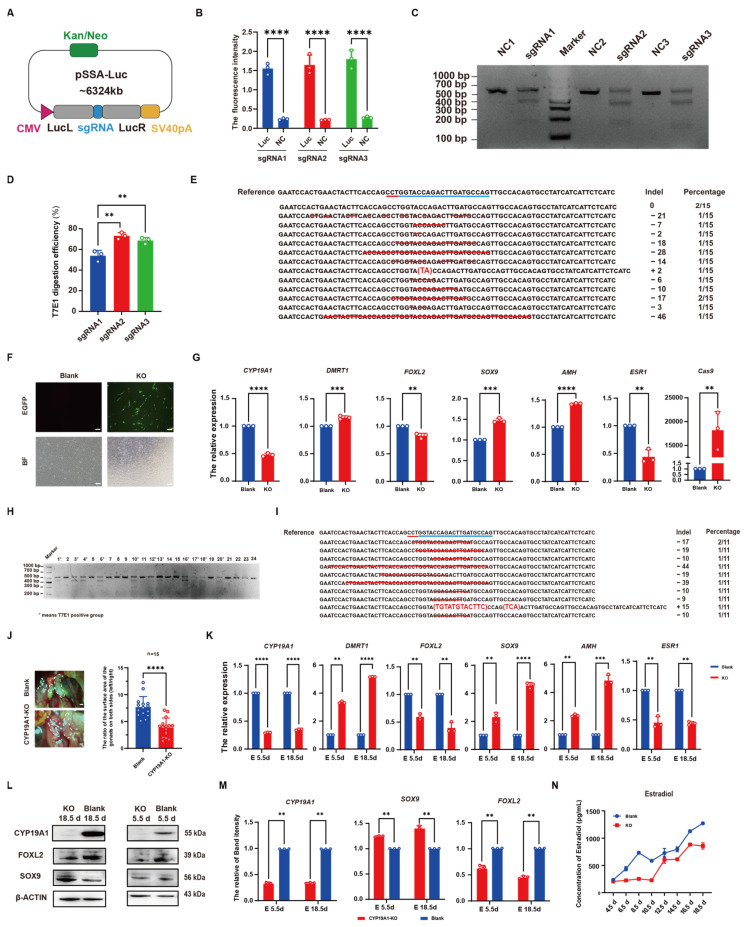
CRISPR/Cas9-mediated effective disruption of *CYP19A1* expression in chicken. (**A**) *CYP19A1*-SSA1, *CYP19A1*-SSA2, *CYP19A1*-SSA3 vector map. (**B**) Luciferase-SSA reporter carrier activity assay. (**C**) *CYP19A1*-sgRNA1, *CYP19A1*-sgRNA2, *CYP19A1*-sgRNA3 T7E1 digestion gel map, M: DL1000. (**D**) Gray value analysis of gel images of *CYP19A1*-sgRNA1, *CYP19A1*-sgRNA2, *CYP19A1*-sgRNA3 T7E1 digestion. (**E**) Detection of *CYP19A1*-KO TA clone. (**F**) Fluorescence image of CEFs transfected with *CYP19A1*-KO vector. Blue underline: Targeted sequence sites for experimental design. Red underline: Protospacer Adjacent Motif (PAM). Red line segment and sequence in parentheses: Deletion and insertion of DNA sequences. (**G**) Fluorescence quantitative expression of sex-related genes and Cas9 after *CYP19A1*-KO vector was transfected into CEF. (**H**) Detection of the digestion of chicken embryo T7E1 after injection of *CYP19A1*-KO vector. (**I**) TA clone of chicken embryos detected by T7E1 enzyme digestion was positive. (**J**) Observation of female morphology and statistics of the ratio of left and right gonadal area. (**K**) Quantitative expression of sex-related genes at 5.5 d and 18.5 d after *CYP19A1*-KO vector injection. (**L**) 5.5 d and 18.5 d after injection of *CYP19A1*-KO vector; the protein expression of sex-related genes. Blue underline: Targeted sequence sites for experimental design. Red underline: Protospacer Adjacent Motif (PAM). Red line segment and sequence in parentheses: Deletion and replacement of DNA sequences. (**M**) Grayscale analysis of the protein expression of sex-related genes at 5.5 d and 18.5 d after *CYP19A1*-KO vector injection. (**N**) Expression of estradiol at different developmental stages after injection of *CYP19A1*-KO vector. (** *p* < 0.01; *** *p* < 0.001; **** *p* < 0.0001).

To assess the knockout activity of the *CYP19A1* knockout vector, three sgRNAs were ligated into the Luciferase-SSA reporter vector. After sequencing, results showed that we successfully constructed the Luciferase-SSA reporter vectors *CYP19A1*-SSA1, *CYP19A1*-SSA2, and *CYP19A1*-SSA3 (Figure 2A). Co-transfection of the knockout vector with the Luciferase-SSA reporter vector and donor vector into cells resulted in significant differences in Luciferase activity compared to the control group, indicating knockout activity of the three sgRNA vectors (Figure 2B). To further select the knockout vector with the highest activity, DF-1 cells were transfected with *CYP19A1*-sgRNA1, *CYP19A1*-sgRNA2, and *CYP19A1*-sgRNA3, respectively, for 48 h, and cells with green fluorescence were collected by flow cytometry for genomic DNA extraction. After PCR amplification of regions flanking the target sites (508 bp for *CYP19A1*-sgRNA1 and *CYP19A1*-sgRNA2, 489 bp for *CYP19A1*-sgRNA3) and T7E1 enzyme digestion, results revealed that three bands were observed in all experimental groups, while a single band was exhibited in the control group, demonstrating knockout activity of the three vectors (Figure 2C). Grayscale analysis showed the knockout efficiencies of 53.93% for *CYP19A1*-sgRNA1, 73.20% for *CYP19A1*-sgRNA2, and 68.64% for *CYP19A1*-sgRNA3 (Figure 2D). These results indicated that *CYP19A1*-sgRNA2 exhibited the strongest knockout activity and highest efficiency, which was selected as the *CYP19A1* knockout vector, designated as *CYP19A1*-KO. TA cloning sequencing showed mutations in 13 out of 15 clones, indicating an 86.6% knockout efficiency for *CYP19A1*-KO (Figure 2E). Transfection of the *CYP19A1*-KO vector into CEF cells for 48 h resulted in green fluorescence expression (Figure 2F). qRT-PCR analysis showed significant downregulation of the female-related genes (*CYP19A1*, *FOXL2*, and *ESR1*) and upregulation of the male-related genes (*DMRT1*, *SOX9*, and *AMH*) in cells transfected with the *CYP19A1*-KO vector compared to the control group (Figure 2G). After the PEI-packaged *CYP19A1*-KO vector was injected into the blunt end of chicken embryos, and after T7E1 enzyme digestion of tissues from 5.5 d and 18.5 d chicken embryos, the results revealed three bands in eleven out of twenty-four experimental chickens, indicating a 46% knockout efficiency for *CYP19A1*-KO in chicken embryos (Figure 2H). The TA cloning sequencing of T7E1-positive embryos showed mutations at the knockout target sites, demonstrating the effective gene deletion by *CYP19A1*-KO in chicken embryos (Figure 2I). Observation of the left and right ovaries of 18.5 d embryos from the control and *CYP19A1* knockout groups revealed a reduction in asymmetrical ovarian development in the *CYP19A1* knockout group, affecting ovarian development (Figure 2J). qRT-PCR analysis of T7E1-positive 5.5 d and 18.5 d embryo gonads showed significant downregulation of female-related genes (*CYP19A1*, *FOXL2*, and *ESR1*) and upregulation of male-related genes (*DMRT1*, *SOX9*, and *AMH*) compared to the control group (Figure 2K). WB results also demonstrated a significant reduction in CYP19A1 and FOXL2 protein levels, and a significant increase in SOX9 protein levels in embryos injected with the *CYP19A1*-KO vector (Figure 2L,M). These results indicated that the *CYP19A1*-KO vector had a functional role in vivo and can influence the expression of sex-related genes. After sex determination was performed on chicken embryos with 4.5 d to 18.5 d post-injection, the gonads of female embryos were collected for Elisa to detect changes in estradiol levels. The results showed a decrease in estradiol levels in the ovaries of embryos injected with *CYP19A1*-KO compared to the normal female gonads (Figure 2N), indicating that the *CYP19A1*-KO vector can affect estrogen expression and, consequently, chicken embryo gender differentiation.

### 3.3. Establishment of CRISPR/Cas9-Mediated Induced Knockout System in Chicken DF-1 Cells

**Figure 3:**
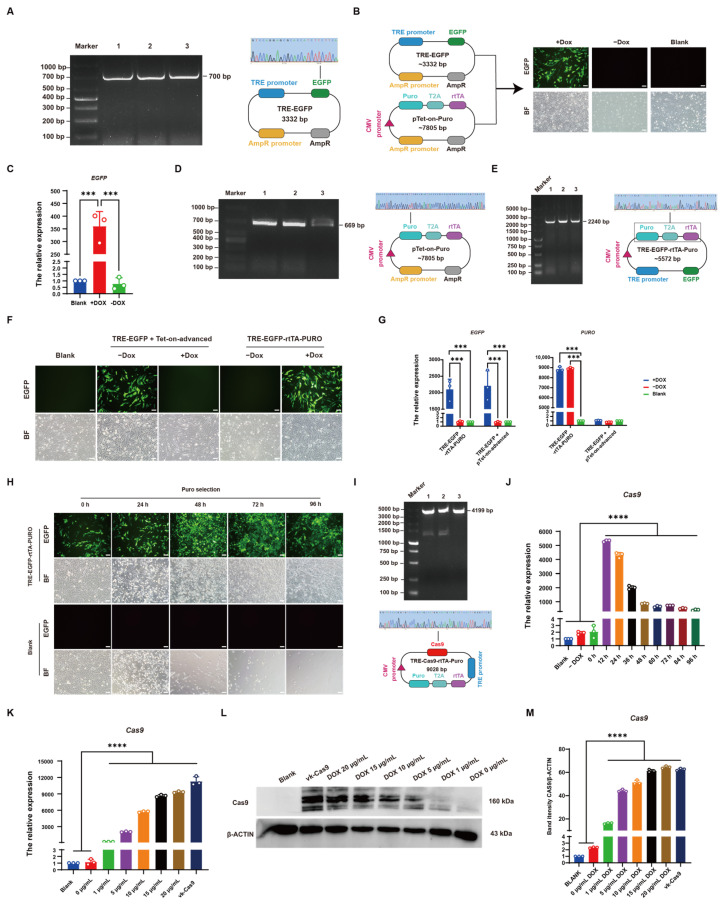
Establishment of CRISPR/Cas9-mediated induced knockout system in chicken DF-1 cells. (**A**) EGFP element amplification fragment gel map and TRE-EGFP carrier sequencing peak map, M: DL1000; 1–3: amplified fragment. (**B**) Fluorescence maps of TRE-EGFP and pTet-on-Puro co transfected cells with and without DOX addition. (**C**) Quantitative expression of EGFP. (**D**) Puro element amplification fragment gel map and pTet-on-Puro vector sequencing peak map, M: DL1000; 1–3: amplified fragment. (**E**) Puro and rtTA element amplification fragment gel map and TRE-EGFP-rtTA-Puro vector sequencing peak map, M: DL5000; 1–3: amplified fragment. (**F**) TRE-EGFP+Tet-on-advanced and TRE-EGFP-rtTA-Puro, respectively, with and without DOX transfection DF-1 fluorescence maps. (**G**) Quantitative expression of EGFP, PURO. (**H**) TRE-EGFP-rtTA-Puro transfection DF-1, 0–96 h Puro screening cell fluorescence map. (**I**) Cas9 element amplification fragment gel map and TRE-Cas9-rtTA-Puro vector sequencing peak map, M: DL5000; 1–4: amplified fragments. (**J**) Quantitative detection of DOX induction at different times. (**K**) Quantitative detection induced by different concentrations of DOX. (**L**) WB detection induced by different concentrations of DOX. (**M**) Grayscale analysis of WB bands. (*** *p* < 0.001; **** *p* < 0.0001).

To validate the functionality of the Tet-on system, the EGFP-N1 vector was used as a template to amplify the EGFP cassette (Figure 3A), which was then ligated into the TRE-tight vector downstream of the TRE promoter. After sequencing, results showed that we successfully constructed the TRE-EGFP vector (Figure 3A). After co-transfection of the TRE-EGFP vector with pTet-On-Advanced into DF-1 cells for 48 h and the addition of 5 μg/mL DOX induction, the results showed that fluorescence was observed in DOX-induced cells, while non-induced cells showed no fluorescence (Figure 3B). qRT-PCR results also showed that a significantly higher EGFP expression was in DOX-induced cells compared to the control group, whereas EGFP expression in non-induced cells had no significant difference from the control (Figure 3C), demonstrating the normal functionality of the Tet-on system. To establish a single-plasmid inducible expression system with selectable markers, the Puro cassette was introduced into the Tet-on system. The Puro cassette was amplified from VK001-08 and ligated into the pTet-on-Advanced vector with the rtTA element linked via a T2A sequence, sharing a single promoter (Figure 3D). Subsequently, the promoter-containing portion of rtTA and Puro elements was amplified from successfully sequenced plasmids (Figure 3D) and ligated into TRE-EGFP, resulting in the successful construction of the TRE-EGFP-rtTA-Puro vector (Figure 3E). After transfection of the vector into DF-1 cells for 48 h and the addition of 5 μg/mL DOX induction, results showed that fluorescence was in DOX-induced cells, while non-induced cells had no fluorescence (Figure 3F). qRT-PCR analysis confirmed that the constructed single-plasmid inducible expression system could induce EGFP expression, with PURO expression significantly increased compared to the control group, regardless of DOX induction (Figure 3G). Transfected cells induced with DOX were subjected to puromycin selection, and after 96 h, nearly all control cells were dead, while only cells expressing green fluorescence survived in transfected cells, demonstrating the functionality of the Puro element for drug screening (Figure 3H). These results collectively confirm the successful construction of the single-plasmid inducible expression system. To further establish an inducible CRISPR/Cas system, the Cas9 element was amplified from VK001-08 (Figure 3I) and replaced the EGFP cassette in TRE-EGFP-rtTA-Puro, resulting in the successful construction of the TRE-Cas9-rtTA-Puro vector (Figure 3I). To determine the optimal induction time for DOX, DF-1 cells were transfected with the vector for 24 h, and 5 μg/mL DOX was added for induction. Cells were collected at 0 h, 12 h, 24 h, 36 h, 48 h, 60 h, 72 h, and 96 h post-induction. qRT-PCR results showed that Cas9 expression was highest at 12 h post-DOX induction (Figure 3J). Additionally, gradient concentrations of DOX (1, 5, 10, 15, 20 μg/mL) were used for induction. qRT-PCR results showed that Cas9 expression increased with increasing concentrations of DOX, with no significant difference in Cas9 expression between 20 μg/mL and Cas9 expression in VK001-08 (Figure 3K). Consistently, WB detection showed similar results (Figure 3L, M). These findings indicate that the optimal induction time for DOX is 12 h, and the optimal concentration is 20 μg/mL.

### 3.4. Establishment of CRISPR/Cas9-Mediated DOX-Induced CYP19A1 Knockout System In Vitro

**Figure 4:**
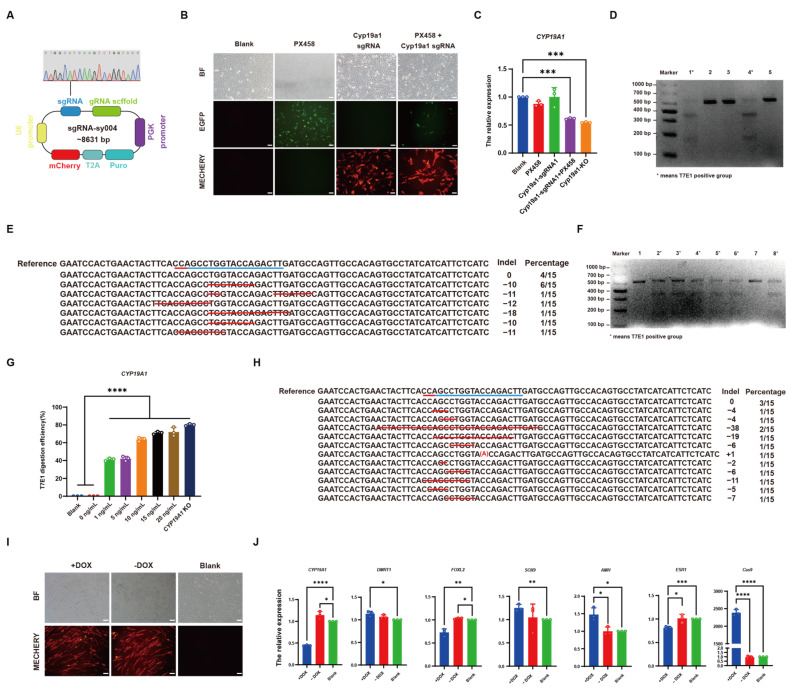
Establishment of CRISPR/Cas9-mediated dox-induced *CYP19A1* knockout system in vitro. (**A**) *CYP19A1*-sgRNA sequencing peaks. (**B**) Fluorescence image of *CYP19A1*-sgRNA co-transfected with PX458. (**C**) Quantitative detection of *CYP19A1*. (**D**) *CYP19A1*-sgRNA T7E1 restriction map. M: DL1000; 1: *CYP19A1*-sgRNA co-transfected with PX458; 2: *CYP19A1*-sgRNA; 3: PX458; 4: *CYP19A1*-KO; 5: DF-1. (**E**) *CYP19A1*-sgRNA TA clone detection. Blue underline: Targeted sequence sites for experimental design. Red underline: Protospacer Adjacent Motif (PAM). Red line segment: Deletion of DNA sequences. (**F**) Inducible *CYP19A1* knockout system T7E1 digestion map. M: DL1000; 1: no DOX induction; 2: 1 μg/mL DOX induction; 3: 5 μg/mL DOX induction; 4: 10 μg/mL DOX induction; 5: 15 μg/mL DOX induction; 6: 20 μg/mL DOX induction; 7: DF-1; 8: *CYP19A1*-KO. (**G**) Grayscale analysis of inducible *CYP19A1* knockout system T7E1 digestion. (**H**) Inducible *CYP19A1* knockout system TA clone detection. Blue underline: Targeted sequence sites for experimental design. Red underline: Protospacer Adjacent Motif (PAM). Red line segment and sequence in parentheses: Deletion and insertion of DNA sequences. (**I**) CEF fluorescence image of transfection-induced *CYP19A1* knockout system. (**J**) Fluorescence quantitative expression of sex-related genes and CAS9 after inducible *CYP19A1* knockout system transfected CEF. (* *p* < 0.05; ** *p* < 0.01; *** *p* < 0.001; **** *p* < 0.0001).

To establish an inducible *CYP19A1* knockout system, sgRNAs from *CYP19A1*-KO were inserted into the sgRNA-sy004 vector. Sequencing confirmed the successful construction of the single sgRNA vector, *CYP19A1* sgRNA (Figure 4A). To assess the knockout activity of the vector, it was co-transfected with the Cas9-containing vector PX458 into DF-1 cells for 48 h. Cells co-transfected with PX458 and CYP19A1 sgRNA vectors exhibited both green and red fluorescence, whereas cells transfected with the CYP19A1 sgRNA vector alone displayed only red fluorescence, and those transfected with the PX458 vector alone exhibited only green fluorescence. (Figure 4B). qRT-PCR revealed that the *CYP19A1* expression level was significantly lower in cells co-transfected with *CYP19A1* sgRNA and PX458 vectors compared to the control group (Figure 4C). Moreover, T7E1 results showed that there were three bands in cells co-transfected with *CYP19A1* sgRNA and PX458 vector, while only one band was observed in cells with single-vector (*CYP19A1* sgRNA or PX458) transfection and the control group (Figure 4D). TA cloning results indicated a knockout efficiency of 74% for *CYP19A1* sgRNA (Figure 4E), confirming its efficacy in guiding knockout. To further select the optimal DOX concentration for inducing the *CYP19A1* knockout system, *CYP19A1* sgRNA was co-transfected with TRE-EGFP-rtTA-Puro into DF-1 cells, followed by induction with gradient concentrations of DOX (1, 5, 10, 15, 20 μg/mL). After 48 h post-induction, T7E1 results revealed three bands in all DOX-induced cells (Figure 4F). As the DOX induction concentration increased, the knockout efficiency also increased. When the DOX concentration reached 20 μg/mL, there was no significant difference between the knockout efficiency and *CYP19A1*-KO knockout efficiency (Figure 4G). TA cloning of cells induced with 20 μg/mL DOX showed that 12 out of 15 colonies were knocked out, with an efficiency of 80%, which is similar to *CYP19A1*-KO (86.6%). These results indicate that 20 μg/mL DOX induces the highest knockout efficiency. To demonstrate the functionality of the inducible *CYP19A1* knockout system in somatic cells, the system was transfected into CEF cells and induced with DOX for 48 h, resulting in fluorescence expression (Figure 4I). qRT-PCR analysis showed that DOX induction suppressed *CYP19A1* expression, subsequently inhibiting the expression of the female-related genes (*FOXL2* and *ESR1*), while significantly increasing the expression of male-related genes (*DMRT1*, *SOX9*, and *AMH*) compared to the control group. The expression levels of gender-related genes has no significant difference between cells without DOX induction and the blank control group (Figure 4J). These results demonstrate that the inducible *CYP19A1* knockout system can successfully knock out the *CYP19A1* gene in vitro with DOX induction, thereby affecting the expression levels of gender-related genes.

### 3.5. CRISPR/Cas9 Effectively Knocked out CYP19A1 via DOX Induction In Vivo

**Figure 5:**
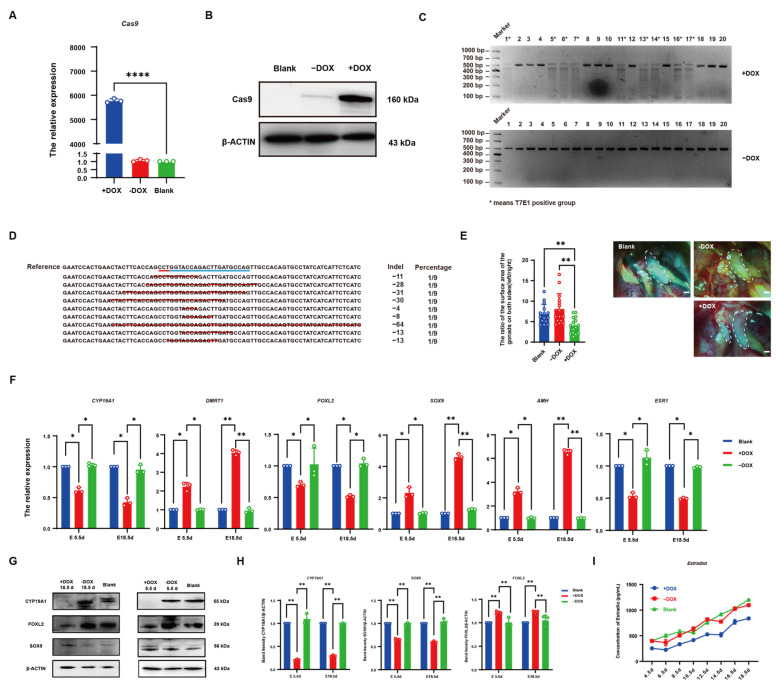
CRISPR/Cas9-mediated effective disruption of *CYP19A1* expression via dox stimulate in oco. (**A**) Quantitative detection of Cas9 at 48 h. (**B**) 48 h Cas9 WB detection. (**C**) Inducible *CYP19A1* knockout dual vector system after injection of chicken embryo T7E1 digestion detection. (**D**) TA clone of chicken embryos detected positive by T7E1 digestion. Blue underline: Targeted sequence sites for experimental design. Red underline: Protospacer Adjacent Motif (PAM). Red line segment: Deletion of DNA sequences. (**E**) Morphological observation of female gonads and statistical analysis of the ratio of left and right gonadal areas. (**F**) 5.5 d and 18.5 d after the injection of *CYP19A1* knockout two-vector system can induce the expression of sex-related genes. (**G**) WB detection of sex-related genes at 5.5 d and 18.5 d after injection of inducible *CYP19A1* knockout dual vector system. (**H**) Grayscale analysis of the protein expression of sex-related genes at 5.5 d and 18.5 d after injection of the inducible *CYP19A1* knockout two-vector system. (**I**) The expression of estradiol at different developmental stages after *CYP19A1* knockout two-vector system can be induced by injection. (* *p* < 0.05; ** *p* < 0.01; **** *p* < 0.0001).

To demonstrate whether the inducible *CYP19A1* knockout system can affect chicken embryo sex differentiation, the PEI-encapsulated inducible *CYP19A1* knockout system and DOX were injected into the blunt end of chicken embryos. After 48 h, chicken embryos were collected, and qRT-PCR results showed that Cas9 expression was significantly higher in DOX-induced embryos compared to the control group, while embryos without DOX induction showed no significant difference in Cas9 expression compared to the control group (Figure 5A). Consistently, WB showed similar results (Figure 5B), confirming that DOX can induce Cas9 expression in vivo. T7E1 enzyme digestion of 5.5-day and 18.5-day chicken embryos showed that three bands were observed in nine out of twenty chicken embryos with DOX induction, demonstrating that the inducible *CYP19A1* knockout system can target and knock out *CYP19A1* in chicken embryos under DOX induction, with a knockout efficiency of 45% (Figure 5C). TA cloning of T7E1-positive chicken embryos showed mutations at the knockout site (Figure 5D). These results indicate that the inducible *CYP19A1* knockout system can effectively mediate gene loss in chicken embryos with DOX induction. For both control and 18.5-day chicken embryos with DOX induction, the surface area ratio of the left and right ovaries was statistically analyzed. It was found that asymmetrical development of the ovaries in female embryos with DOX induction was attenuated compared to those without DOX induction, while asymmetrical development was observed in the embryos without DOX induction, indicating that the *CYP19A1* knockout system affects ovarian development (Figure 5E). qRT-PCR analysis of T7E1-positive chicken embryos from both 5.5-day and 18.5-day DOX-induced groups showed significant downregulation of female-related genes (*CYP19A1*, *FOXL2*, and *ESR1*) and significant upregulation of male-related genes (*DMRT1*, *SOX9*, and *AMH*) compared to the control group, while there was no significant difference in the expression levels of gender-related genes in embryos without DOX induction compared to the control group (Figure 5F). WB results showed that the CYP19A1 protein expression level decreased in 5.5-day and 18.5-day chicken embryos after DOX induction, concurrently inhibiting the protein expression level of the female-related gene, *FOXL2*, and promoting the protein expression level of the male-related gene, *SOX9*, whereas there was no significant change in the embryos without DOX induction (Figure 5G,H). These findings demonstrate that the inducible *CYP19A1* knockout system, with DOX induction, can knock out *CYP19A1* in vivo and further affect the expression level of gender-related genes. The Elisa analysis of the PEI-injected 4.5-day to 18.5-day female chicken embryos’ ovaries showed that estradiol levels in DOX-induced embryos were significantly lower than those in normal female ovaries and the embryos without DOX induction (Figure 5I). These results indicate that the inducible *CYP19A1* knockout system, with DOX induction, can knock out *CYP19A1* in vivo and further affect the estradiol expression level.

## 4. Discussion

Despite decades of research on avian sex differentiation, its molecular mechanisms remain elusive. In this study, the crucial role of *CYP19A1* in female sex differentiation in chickens was successfully validated. Additionally, an inducible *CYP19A1* knockout chicken model was established using CRISPR/Cas9 technology, allowing for a systematic investigation of the role of *CYP19A1* in sex differentiation during embryonic development in *Gallus gallus*. Unlike mammals with symmetrical ovarian development, birds lose the right ovary and oviduct during sexual development [26]. *CYP19A1* shows specific expression in early female embryos before the onset of ovarian differentiation and is expressed in ovarian medullary cells [27,28,29]. These results demonstrate the crucial role of *CYP19A1* in female chicken embryo gonadal sex differentiation.

In Chinese soft-shelled turtles, the overexpression or interference of *CYP19A1* leads to sex reversal, with overexpression inducing ovarian development in males [30]. In African clawed frogs, the knockout of *CYP19A1* results in all-male offspring, highlighting the importance of *CYP19A1* in female gonadal differentiation [31]. During the bipotential stage of chicken embryo gonads, treatment with aromatase inhibitors leads to the development of permanent male phenotypes in genetic females, accompanied by decreased expression of the female-specific gene *FOXL2* in the ovaries, demonstrating the importance of P450arom in female sex differentiation [32]. These data clearly indicate that *CYP19A1* is a key gene in chicken female gonadal differentiation, capable of initiating male gonadal differentiation and inhibiting female gonadal differentiation.

Previous experiments on *CYP19A1* gene overexpression and interference showed that overexpression promotes the development of female gonads, while interference leads to female gonad differentiation towards male gonads. Therefore, three knockout target sites for *CYP19A1* were designed, and the most efficient target site was identified through a series of tests. TA cloning results showed an 80% knockout efficiency at this site in chicken fibroblasts and a 45% knockout efficiency in vivo, accompanied by decreased expression of gender-related genes and estradiol levels. These results indicate that the knockout of *CYP19A1* directly affects the expression of sex-related genes, such as the upregulation or downregulation of *FOXL2*, *ESR1*, *DMRT1*, *SOX9*, and *AMH* genes, thereby altering the process of sex differentiation. In particular, in female chicken embryos, the downregulation of *FOXL2* and *ESR1*, along with the upregulation of male-related genes *DMRT1*, *SOX9*, and *AMH*, suggests that female gonadal differentiation is suppressed, while male gonadal differentiation is induced. This further supports the regulatory role of *CYP19A1* in sex differentiation. Moreover, changes in hormone levels represent another key finding of this study. ELISA results revealed that, in DOX-induced female chicken embryo gonads, the levels of estradiol were significantly reduced. Estradiol, the main estrogen synthesized by *CYP19A1*, plays a critical role in the development of female gonads. The decrease in estradiol levels indicates that the knockout of *CYP19A1* significantly reduces estrogen synthesis, thus affecting the development of female gonads. This finding further reinforces the central role of *CYP19A1* in sex differentiation, particularly in regulating the synthesis of sex hormones. This demonstrates the successful establishment of a *CYP19A1* knockout system that can be used to establish a chicken sex control model.

At the transcriptional level, the Tet-on system has become one of the methods for inducible control of CRISPR/Cas9 [33]. Daichi et al. combined CRISPR/Cas and the Tet-on system for spatiotemporal control of targeted gene expression in zebrafish, but limited space may result in an insufficient DOX supply to experimental fish, and adding more DOX particles may increase the risk of increasing the DOX concentration [34]. In contrast, combining CRISPR/Cas9 and the Tet-on system in chickens to construct a Tet-on-induced CRISPR/Cas9 system allows relatively precise control of DOX concentration when the drug is added, minimizing environmental interference. The results of this study show that under DOX induction at 20 μg/mL, the inducible *CYP19A1* knockout system can efficiently achieve targeted mutations in chicken *CYP19A1*. Currently, the in vivo knockout efficiency in this study is 45%, which has not yet achieved 100% control. The inducibility of the Tet-on system still has certain limitations; however, further optimization of the DOX concentration and administration methods (such as chronic administration or local injection) could enhance the inducibility of the system, thereby improving knockout efficiency. Existing delivery vectors may also present issues with insufficient delivery efficiency. Future research could focus on developing novel delivery methods, such as nanoparticles or electroporation techniques, to increase gene editing efficiency and, consequently, improve knockout efficiency, while further optimizing the inducibility and specificity of the system.

## 5. Conclusions

In summary, our results demonstrate that *CYP19A1* is a key factor in chicken gonadal development and sex differentiation. Additionally, this study successfully constructed an inducible *CYP19A1* knockout system using the Tet-on-induced CRISPR/Cas9 system. Through in vitro and in vivo validation, we have shown that, under DOX induction, efficient knockout of *CYP19A1* can be detected not only in chicken DF-1 cells, but also in the chicken body. This provides strong evidence for the successful application of CRISPR/Cas9 in chickens and lays the foundation for establishing a chicken sex control model. The successful establishment of this inducible knockout system is a significant step forward in avian sex differentiation research and provides an effective tool for sex control in poultry.

## Figures and Tables

**Table 1:** Summary of sgRNAs sequences.

sgRNA Type	Target Region	Sequences: 5′-3′	PAM
sgRNA 1	Exon 1	CATCAAGTCTGGTACCAGGC	TGG
sgRNA 2	Exon 1	CTGGCATCAAGTCTGGTACC	AGG
sgRNA 3	Exon 3	CATTACGTGAAATACGCTGG	AGG

**Table 2:** PCR primer sets.

Gene	Primer Sequences (5′-3′)
*CYP19A1-1*	F: CTCTTAGTCCATCAAGTCTGGTACCAGGC R: CTCTAAAACGCCTGGTACCAGACTTGATG
*CYP19A1-2*	F: CTCTTAGTCCTGGCATCAAGTCTGGTACC R: CTCTAAAACGGTACCAGACTTGATGCCAG
*CYP19A1-3*	F: CTCTTAGTCCATTACGTGAAATACGCTGG R: CTCTAAAACCCAGCGTATTTCACGTAATG
*β-ACTIN*	F: CAGCCATCTTTCTTGGGTAT R: CTGTGATCTCCTTCTGCATCC
*AMH*	F: GAAGCATTTTGGGGACTGG R: GGGTGGTAGCAGAAGCTGAG
*SOX9*	F: AAGGAGAGCGACGAGGACAAA R: GGTCCAGTCGTAGCCCTTGA
*FOXL2*	F: CCTCAACGAGTGCTTCATCA R: ACATCTGGCAAGAGGCGTAG
*ESR1*	F: TAGCCTGAAGCATAGTCGTT R: TTTGGGATGAGGGAAGC
*CYP19A1*	F: CTCCAGCAGGTTGAAGGTA R: ATGAGAATGATGATAGGCAC
*DMRT1*	F: CCCTGCTCACTCCACG R: CACTGGTTTCCTCCCT
*Cas9*	F: GGACATCGGCACCAAC R: CTCGTGCTTCTTATCCTCTT
*Puro*	F: CGCAGCAACAGATGGAA R: TCGGCGGTGACGGTGAA
*CYP19A1sg1*	F: CAGCAGGTTGAAAGGTAC R: GCATATACACAATAGAAGCAAAAC
*CYP19A1sg2*	F: CAGCAGGTTGAAAGGTAC R: GCATATACACAATAGAAGCAAAAC
*CYP19A1sg3*	F: CATAATAGAAGCAACAATGTC R: GTGAGAGCATGTTTTTTTTG
*EGFP-1*	F: CCGGAATTCCGCCACCATGGTGAGCAAG T R: CGCGGATCCTTACTTGTACAGCTCGTCCATGC
*Puro-1*	F: CCGGAATTCATGACCGAGTACAAGCCC R: GCTCTAGACATTGGGCCAGGATTCTC
*Puro+rtTA-1*	F: ACATGTGACATTGATTATTGACTAGTTATTAATAG R: ACATGTAACTTGTTTATTGCAGCTTATAATG
*Cas9-1*	F: CGGGGTACCCCCCAAAGAAGAAGCGGAAG R: ATTTGCGGCCGCTTTCTTTTTCTTAGCTTGACCAGC

Note: The underlined line indicates the restriction site in primer. GAATTC is EcoRI, GGATCC is BamHI, TCTAGA is XbaI, GGTACC is KpnI, GCGGCCGC is NotI, ACATGT is PciI.

## Data Availability

The data that support the findings of this study are available from the corresponding author upon reasonable request.

## Supplementary Materials

The following supporting information can be downloaded at: https://www.mdpi.com/article/10.3390/vetsci12040296/s1, Figures: The original images of Western Blots.
