# Supplementary material for: An Inducible CYP19A1 Excision Model for Sexual Differentiation in Chicken (Gallus gallus) via the CRISPR/Cas9 System
Source: Vet Sci. 2025 Mar 22;12(4):296. doi: 10.3390/vetsci12040296 (PMC12031565; doi:10.3390/vetsci12040296)

Figure 1D Cas9

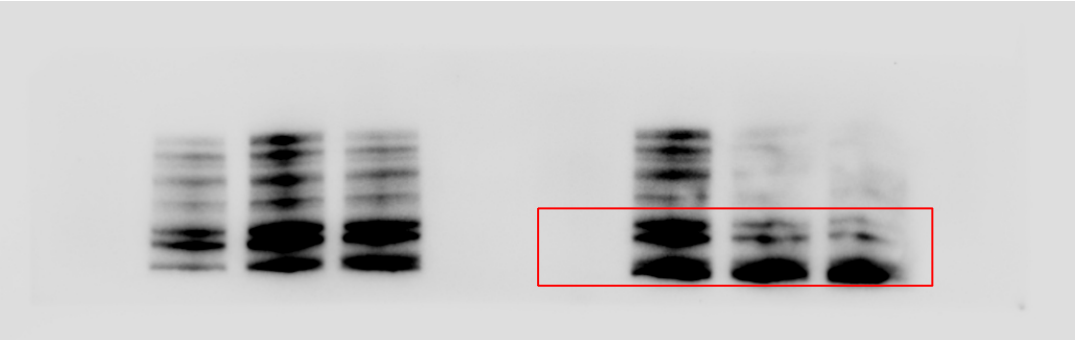

Figure 1D EGFP

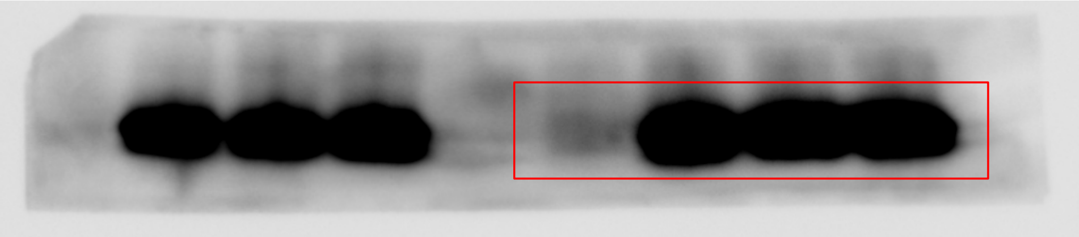

Figure 1D β-actin

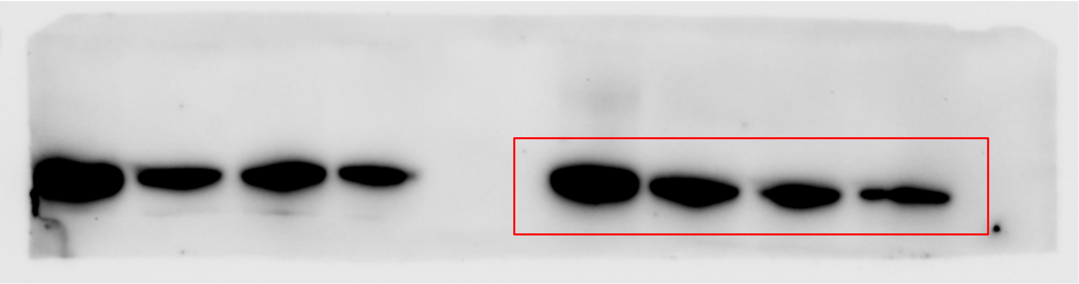

Figure 2C T7E1

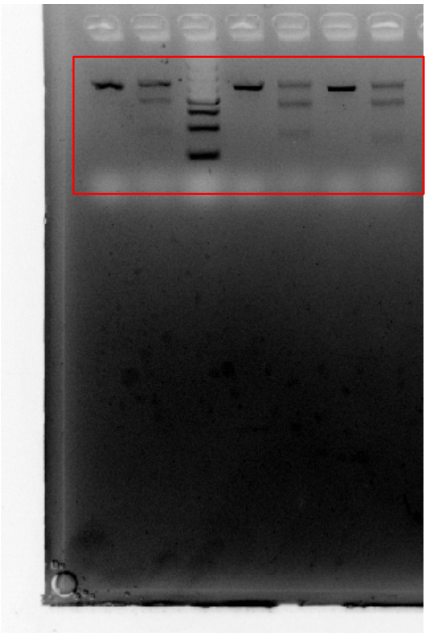

Figure 2H T7E1

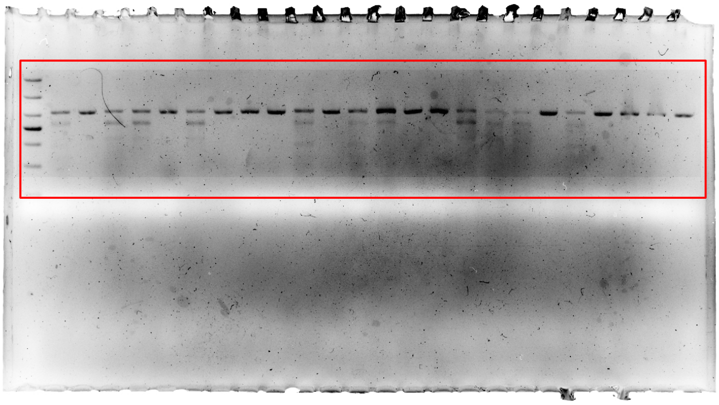

Figure 3A

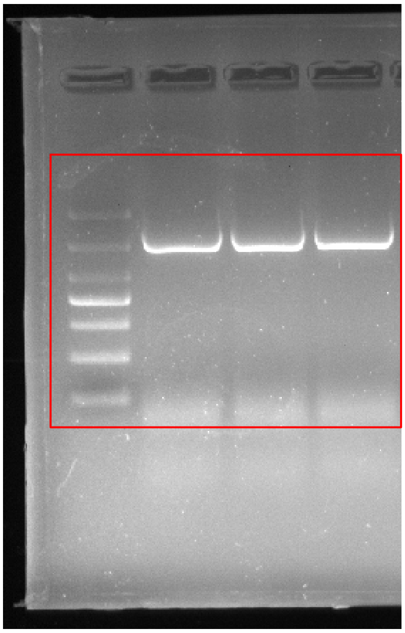

Figure 3D

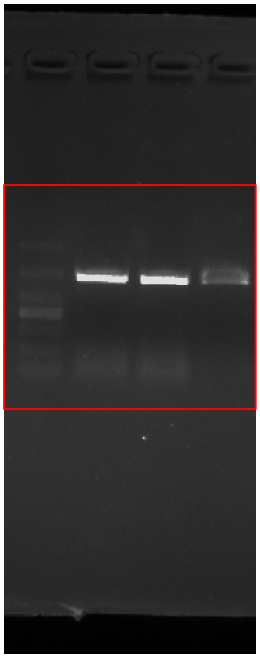

Figure 3E

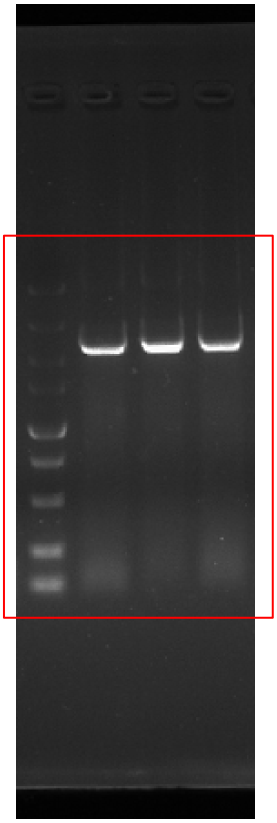

Figure 3I

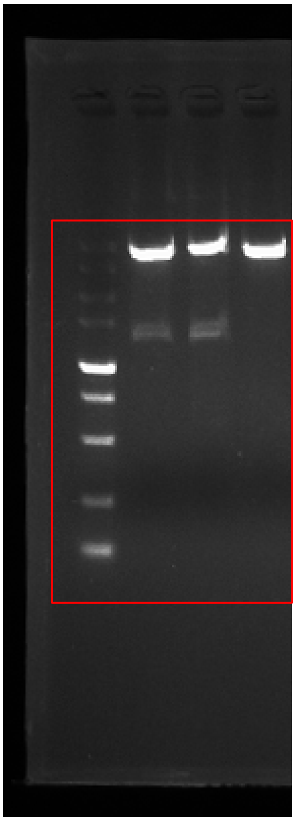

Figure 3L Cas9

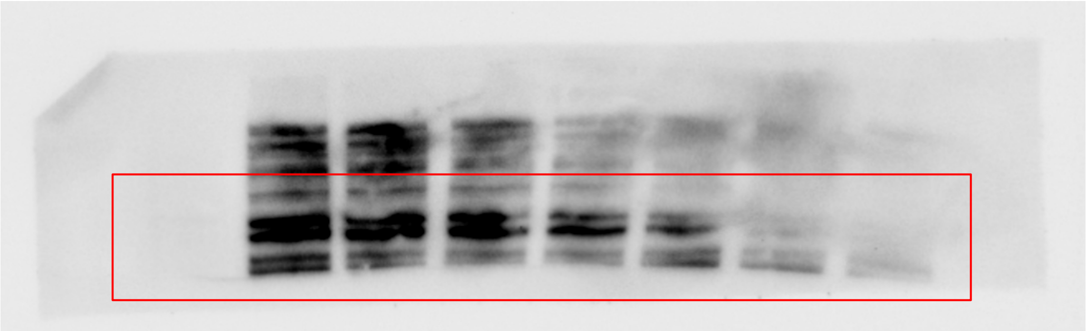

Figure 3L β-ACTIN

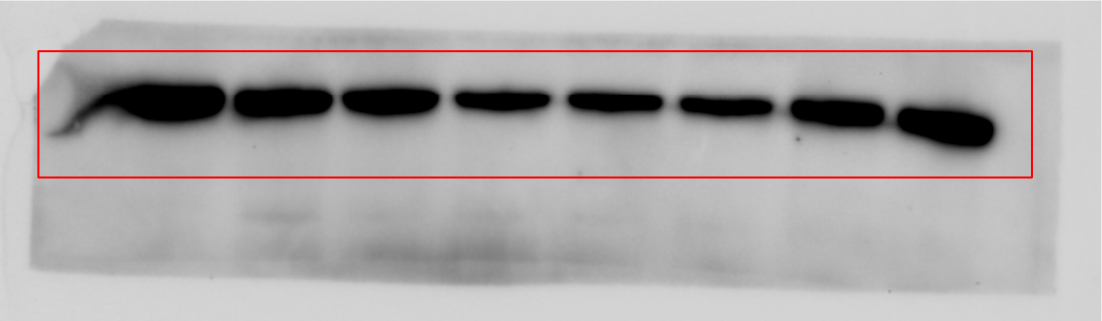

Figure 4D T7E1

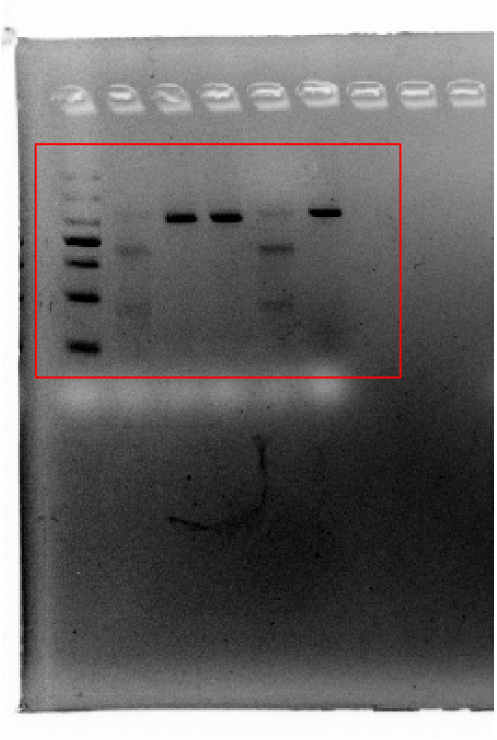

Figure 4F T7E1

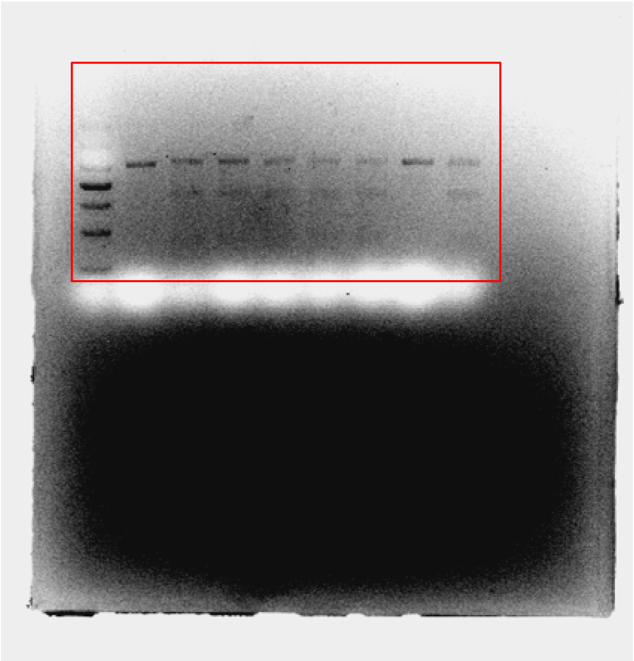

Figure 5B Cas9

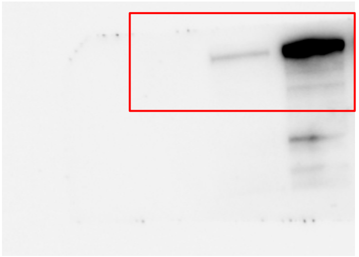

Figure 5B β-ACTIN

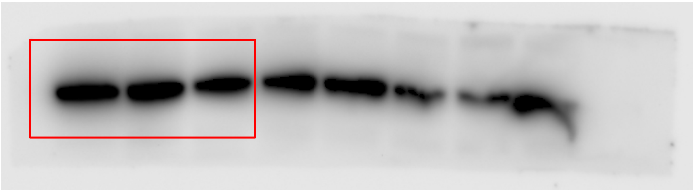

Figure 5C

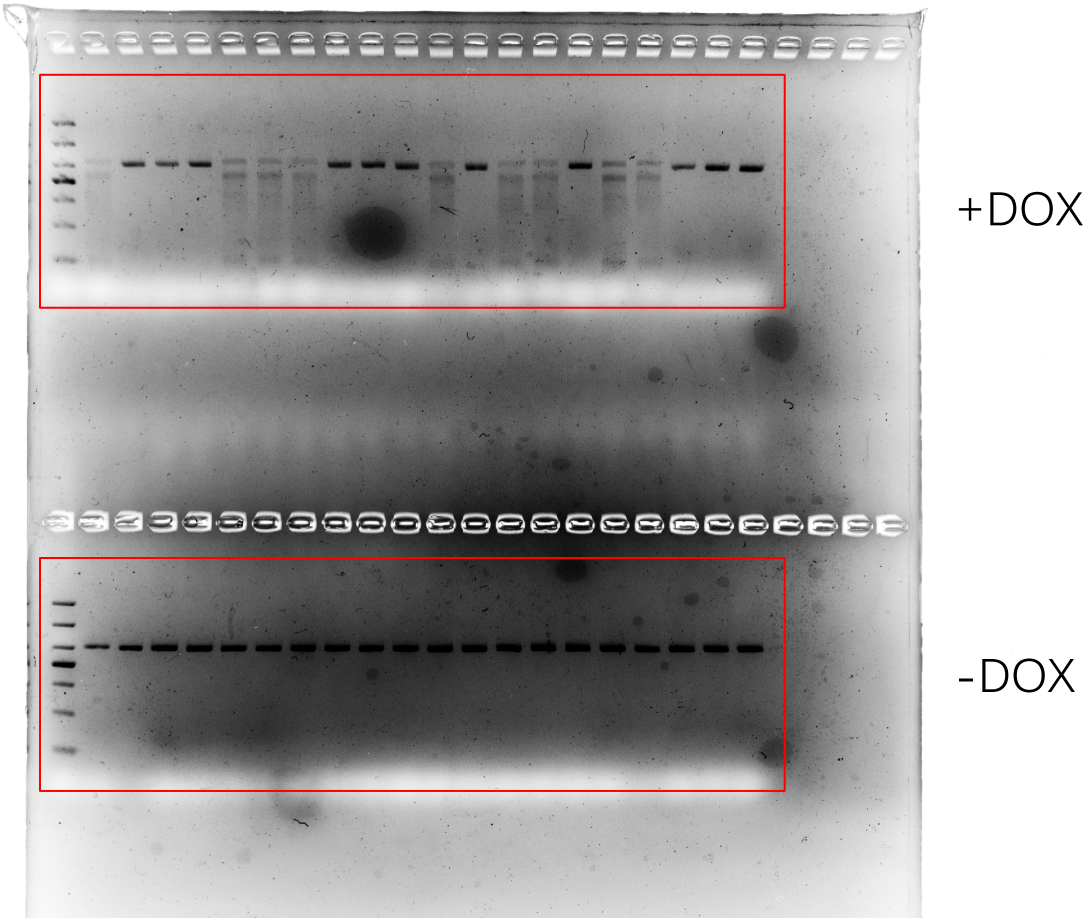

Supplement: Supplementary file 1 [file vetsci-12-00296-s001.zip › vetsci-3326158-supplementary.pdf]
